# Proteomic Analysis of *Pseudomonas putida* Reveals an Organic Solvent Tolerance-Related Gene *mmsB*

**DOI:** 10.1371/journal.pone.0055858

**Published:** 2013-02-11

**Authors:** Ye Ni, Liang Song, Xiaohong Qian, Zhihao Sun

**Affiliations:** The Key Laboratory of Industrial Biotechnology, Ministry of Education, School of Biotechnology, Jiangnan University, Wuxi, Jiangsu, People’s Republic of China; Uni. of South Florida, United States of America

## Abstract

Organic solvents are toxic to most microorganisms. However, some organic-solvent-tolerant (OST) bacteria tolerate the destructive effects of organic solvent through various accommodative mechanisms. In this work, we developed an OST adapted strain *Pseudomonas putida* JUCT1 that could grow in the presence of 60% (v/v) cyclohexane. Two-dimensional gel electrophoresis was used to compare and analyze the total cellular protein of *P. putida* JUCT1 growing with or without 60% (v/v) cyclohexane. Under different solvent conditions, five high-abundance protein spots whose intensity values show over 60% discrepancies were identified by MALDI-TOF/TOF spectra. Specifically, they are arginine deiminase, carbon-nitrogen hydrolase family putative hydrolase, 3-hydroxyisobutyrate dehydrogenase, protein chain elongation factor EF-Ts, and isochorismatase superfamily hydrolase. The corresponding genes of the latter three proteins, *mmsB*, *tsf*, and *PSEEN0851*, were separately expressed in *Escherichia coli* to evaluate their effect on OST properties of the host strain. In the presence of 4% (v/v) cyclohexane, *E. coli* harboring *mmsB* could grow to 1.70 OD_660_, whereas cell growth of *E. coli* JM109 (the control) was completely inhibited by 2% (v/v) cyclohexane. Transformants carrying *tsf* or *PSEEN0851* also showed an increased resistance to cyclohexane and other organic solvents compared with the control. Of these three genes, *mmsB* exhibited the most prominent effect on increasing OST of *E. coli*. Less oxidation product of cyclohexane was detected because *mmsB* transformants might help keep a lower intracellular cyclohexane level. This study demonstrates a feasible approach for elucidating OST mechanisms of microorganisms, and provides molecular basis to construct organic-solvent-tolerant strains for industrial applications.

## Introduction

Whole-cell catalyzed reactions offer many advantages over those catalyzed by isolated enzymes. Biocatalytic processes involving multiple enzymes or co-factor regeneration are more feasible with whole-cell system. Additionally, the intracellular environment of microbial cells (such as pH, temperature, and ionic concentration) is usually favorable for enzymatic activity and stability [1]. However, aqueous/organic solvent biphasic system is often introduced in biotransformation reactions for improved substrate/product solubility, reduced inhibitory effect, as well as easier product recovery [2]. In an octanol/water two-phase reaction system equipped with hollow-fiber membrane, 3-methylcatechol is produced from toluene by an organic-solvent-tolerant (OST) *Pseudomonas putida* [3], and similarly, bioconversion of glucose to phenol is achieved in a biphasic system catalyzed by *P. putida* cells [4]. In our previous study, a dibutyl phthalate/water biphasic system was used to produce (*R*)-2-hydroxy-4-phenylbutyrate by *Candida krusei* [5]. Most organic solvents used in reaction system are toxic to microbial cells and could compromise their viability. Organic solvent molecules disrupt the lipid bilayer of cell membrane, and thus break the structural and functional integrity of cells. The accumulation of various solvents in cell membrane is a major cause for the toxicity of organic solvents, which are often structurally unrelated [6], [7]. Some microorganisms can assimilate toxic organic solvents when the solvent concentrations are low [8], [9]. For some organic solvents, toluene for example, concentration as low as 0.1% (v/v) was toxic enough to microbial cells. It is therefore important to understand the mechanisms of OST in microorganisms for the development of solvent-tolerant strains of industrial interest.

Since the first OST *Pseudomonas putida* strain was isolated in 1989 [10], OST mechanisms of microorganisms have been investigated over the past two decades. In *P. putida*, cell membrane fluidity and solvent tolerance could be adjusted by compositions of membrane lipids and lipopolysaccharide [11]. Isken and de Bont first reported an energy-dependent toluene export system in *P. putida* [12]. Later, three toluene efflux pumps (TtgABC, TtgDEF, and TtgGHI), energy transduction complex (TonB), as well as flagellum biosynthesis genes were proven to be correlated with innate solvent tolerance of *P. putida* [13]–[17].

Several OST *Escherichia coli* mutants were obtained by spontaneous or nitrosoguanidine (NTG) mutations [18]. Similar OST mechanisms (such as membrane properties and efflux pumps) also exist in *E. coli*. A low-level adherence of solvent to OST *E. coli* cells was observed due to their less hydrophobic cell surface than parent strains [19]. As a probable member of *mar-sox* regulon, outer-membrane protein *TolC* is a portion of *AcrAB-TolC* solvent-extruding pump and plays an important role in maintaining and elevating the OST of *E. coli* [20], [21].

In order to elucidate the microbial OST mechanisms, Hayashi and Shimizu et al. investigated the gene expression profiling by DNA microarray. Membrane-associated proteins FruA and GlpC were recognized as OST-related proteins, which may change the cell surface properties and reduce the hydrophobicity of cytomembrane [22], [23]. Additionally, the over-expression of *purR* and *manXYZ* could result in increased solvent-tolerance of *E. coli* JA300, whereas the individual expression of *manX*, *manY*, or *manZ* has no such effect [24], [25].

Two-dimensional gel electrophoresis (2-DE) is commonly used to characterize proteomic differences associated with progression of certain phenotype. In proteomics analysis of *Pseudomonas putida*, Segura and Volkers et al. identified several energy transport and stress-related proteins (CspA, XenA, ATP synthase, etc.) which participate in OST response [26], [27]. In this study, proteomics analysis using 2-DE was also adopted in the exploration of microbial solvent tolerance.

Here, we used 2-DE to compare and analyze the total cellular protein of an OST adapted strain *P. putida* JUCT1 growing with or without organic solvent cyclohexane. We also identified protein spots with significantly enhanced expression level by MALDI-TOF/TOF spectra. It is confirmed that three genes (*mmsB*, *tsf*, and *PSEEN0851*) contribute to the OST phenotype of *P. putida*. The function of these genes suggests that OST mechanisms in microbial cells could also be related to *mar-sox* regulon, some undefined stress-response mechanism, and amino acids metabolic pathways.

## Results

### Adaptation of *P. putida* in Cyclohexane

**Figure 1:**
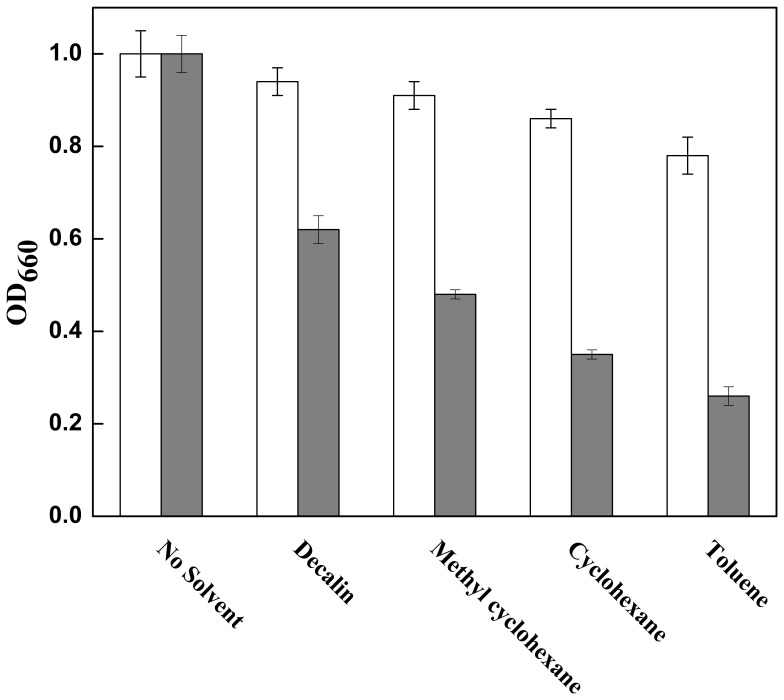
Effect of organic solvents on cell growth of *P. putida* JUCT1 (open bar) and its parent strain JUCS (gray bar). The strains were initially grown in nutrient medium at 37°C till OD_660_ reached 0.2, and then 60% (v/v) organic solvent was added for further incubation of 5 h.

An OST strain *P. putida* JUCT1 was obtained through gradient adaptation in medium containing cyclohexane over 12 serial transfers. Fig. 1 shows the tolerant level of *P. putida* JUCT1 towards various organic solvents including decalin (Log *P*_ow_ = 4.8), methyl cyclohexane (Log *P_ow_* = 3.7), cyclohexane (Log *P_ow_* = 3.2), and toluene (Log *P_ow_* = 2.5). Log *P_ow_* is defined as the common logarithm of a partition coefficient (*P_ow_*) of the solvent between *n*-octanol and water, and is regarded as an index of the solvent toxicity for most microorganisms [6], [10]. Compared with cell growth in medium without solvent (1.0 OD_660_ for both *P. putida* JUCT1 and JUCS), OST adapted strain *P. putida* JUCT1 could grow well in the presence of 60% (v/v) of all solvents tested. Cell densities (OD_660_) of 0.94 (decalin), 0.91 (methyl cyclohexane), 0.86 (cyclohexane) and 0.78 (toluene) were attained after 5-h incubation in various solvents. In contrast, the growth of parent strain *P. putida* JUCS was almost restrained by high concentration of toluene (OD_660_ increase <0.1), while around 0.15 to 0.45 OD_660_ increase was observed with decalin, methyl cyclohexane and cyclohexane. Our results also indicate that the Log *P_ow_* of organic solvent is closely related to their inhibition on cell growth, the lower the Log *P_ow_* value, the higher the toxicity of the solvent [9], [10].

### 2-DE Analysis of Total Cellular Protein of *P. putida* JUCT1

**Figure 2:**
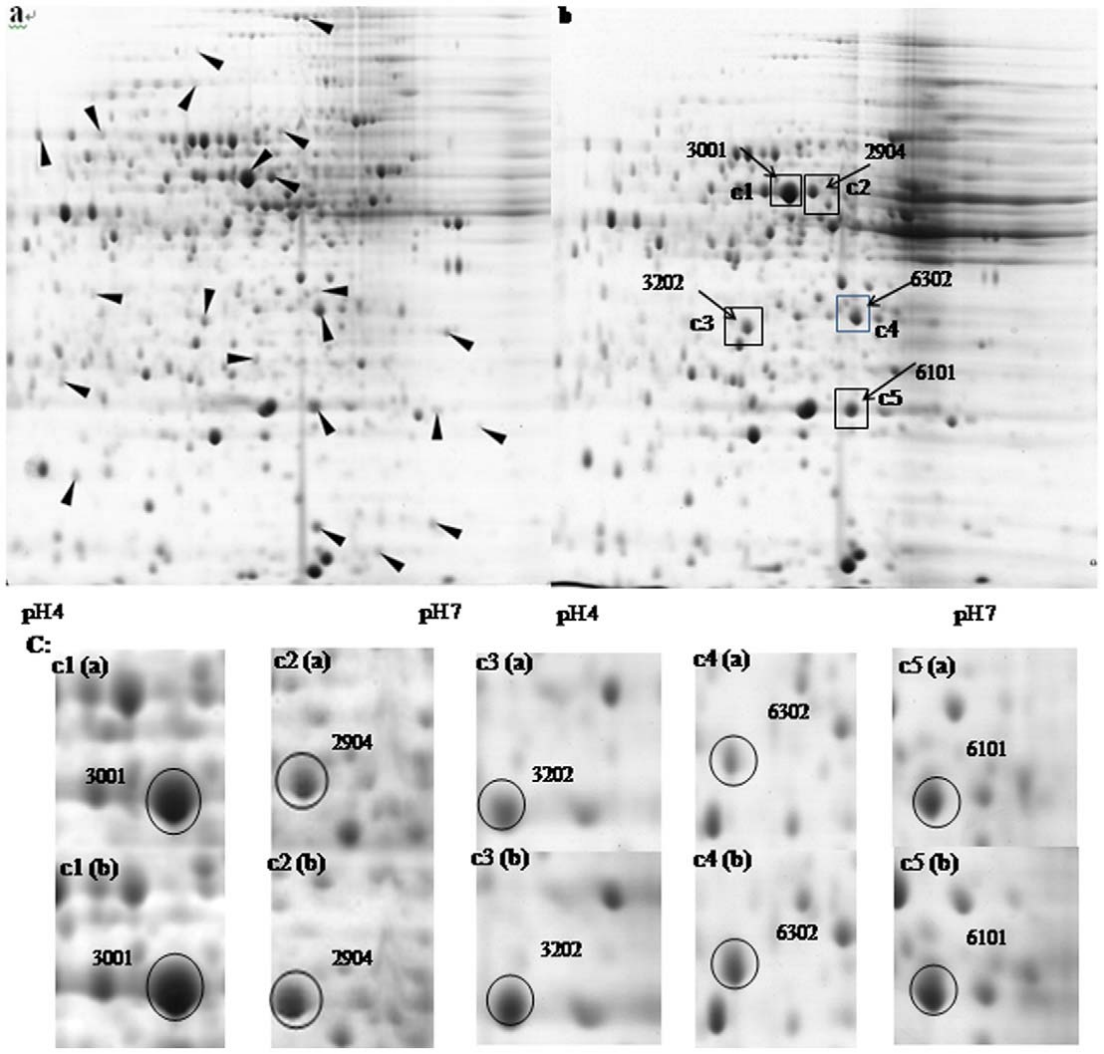
2-DE images of protein extracts of *P. putida* JUCT1 grown under different solvent conditions. a: nutrient medium without solvent; b: with 60% (v/v) cyclohexane; c: magnification of c1–c3, the protein spots selected in this study are circled. Arrowheads indicate the protein spots exhibiting intensity discrepancy of over 50% in samples a and b.

Various chaotropes, surfactants, and reducing agents were added to extract the total cellular protein of *P. putida* JUCT1, and about 6 mg protein was extracted from 0.1 g wet cells. From the image of 2-D gels, 486 spots were detected (Fig. 2). The quantity of total protein spots is similar to *P. putida* UW4 in a previous report [28]. In total cellular protein of *P. putida* JUCT1 grown in the presence of 60% (v/v) cyclohexane (Fig. 2b), the expression level of 22 proteins were detected to be significantly higher than their counterpart without solvent (Fig. 2a), showing over 50% discrepancies in intensity values between two samples. In the 2-DE images, a majority of 22 protein spots are low-abundance proteins, and 5 high-abundance proteins whose expression levels were up-regulated for over 60% were chosen for further study (Fig. 2b, 2c).

### Protein Identification by MALDI-TOF/TOF

Five high-abundance proteins were in-gel digested and analyzed by MALDI-TOF/TOF spectra based on search against NCBI database. For each protein, 1−4 unique matching peptide was observed, and over 60% of amino acids sequences were covered by matching peptides (7−24 peptides/protein) (Table 1). According to above stringent database comparison, five proteins match corresponding *Pseudomonas* proteins: arginine deiminase, carbon-nitrogen hydrolase family putative hydrolase, 3-hydroxyisobutyrate dehydrogenase, protein chain elongation factor EF-Ts, and isochorismatase superfamily hydrolase, which are encoded by *arcA* (GenBank: CAK17111), *PSEEN1080* (GenBank: CAK13980), *mmsB* (GenBank: JC7926), *tsf* (GenBank: CAK16908), and *PSEEN0851* (GenBank: CAK13762) respectively (Table 2).

**Table 1:** MALDI-TOF/TOF analysis of peptides from 5 high-abundance proteins.

SSP	Protein	No. ofpeptides^a^	No. of uniquepeptides^b^	Total sequence^c^	Sequencecoverage (%)^d^
3001	arginine deiminase	24	3	418	68.6
2904	putative hydrolase, carbon-nitrogen hydrolase family	10	2	263	73.1
3202	3-hydroxyisobutyrate dehydrogenase	11	1	295	64.8
6302	proteinchain elongation factor EF-Ts	19	4	287	81.5
6101	Isochorismatase superfamily hydrolase	7	1	206	67.2

a Number of peptides identified for individual protein in MALDI-TOF/TOF analysis.

b Number of unique peptides identified for individual protein.

c Total amino acid sequence of peptides identified for individual protein.

d Percentage of amino acid sequence covered by peptides of individual protein.

**Table 2:** Five high-abundance protein spots identified by MALDI-TOF/TOF.

SSP	Protein	Gene	Intensity values of spot	
			A^a^	B^b^
3001	arginine deiminase	*arcA*	21910.2±174.6	36522.2±298.7
2904	putative hydrolase, carbon-nitrogen hydrolase family	*PSEEN1080*	6575.6±34.1	12626.5±139.6
3202	3-hydroxyisobutyrate dehydrogenase	*mmsb*	4699.5±46.4	11110.1±66.9
6302	proteinchain elongation factor EF-Ts	*tsf*	8314.4±51.7	14962.7±102.4
6101	Isochorismatase superfamily hydrolase	*PSEEN0851*	8520.3±32.6	15962.7±82.2

a The expression level of *P. putida* JUCT1 grown in nutrient medium without solvent.

b The expression level of *P. putida* JUCT1 grown in the presence of 60% (v/v) cyclohexane.

Arginine deiminase pathway is known as a major energy source in many bacteria and archaea. In accordance with our study, the expression level of arginine deiminase was also enhanced in the proteomics analysis of *Pseudomonas putida* DOT-T1E under toluene shock, and the elevated arginine deiminase is presumed to be helpful for generating enough energy to repel organic solvents [26]. Carbon-nitrogen hydrolase family putative hydrolase, sharing over 90% similarity with amino acid sequence of nitrilase/cyanide hydratase and apolipoprotein N-acyltransferase (YP_001667192) from *Pseudomonas putida* GB-1, has not been previously reported in OST-related mechanism, and its role in the solvent response of *P. putida* is currently under investigation. 3-Hydroxyisobutyrate dehydrogenase is essential for valine metabolism, as well as the degradation of leucine and isoleucine. It catalyzes the reversible oxidation of L-3-hydroxyisobutyrate to methylmalonate semialdehyde [29]. Protein chain elongation factor EF-Ts plays an important role in the protein translation process, and catalyzes the regeneration of EF-Tu-GDP complex. Similarly, translation-related proteins have been identified to respond to toluene stress in *P. putida* strains DOT-T1E and S12, such as translation elongation factor Tuf-1 [26] and elongation factor TufB [27]. The function and mechanism of isochorismatase superfamily hydrolase are however still unclear [30]. In this study, the latter three genes, *mmsB, tsf, and PSEEN0851*, were further investigated for their roles in microbial OST.

### Solvent Tolerance of *mmsB, tsf, and PSEEN0851* Transformants

DNA sequencing analysis demonstrates that gene sequence of the open reading frame (including promoter) of *mmsB*, *tsf*, and *PSEEN0851* from *P. putida* JUCT1 are completely the same as those from its parent strain, indicating that no mutation was occurred, and the enhanced expression level of these genes is mainly responsible for the solvent tolerance under cyclohexane condition.

**Figure 3:**
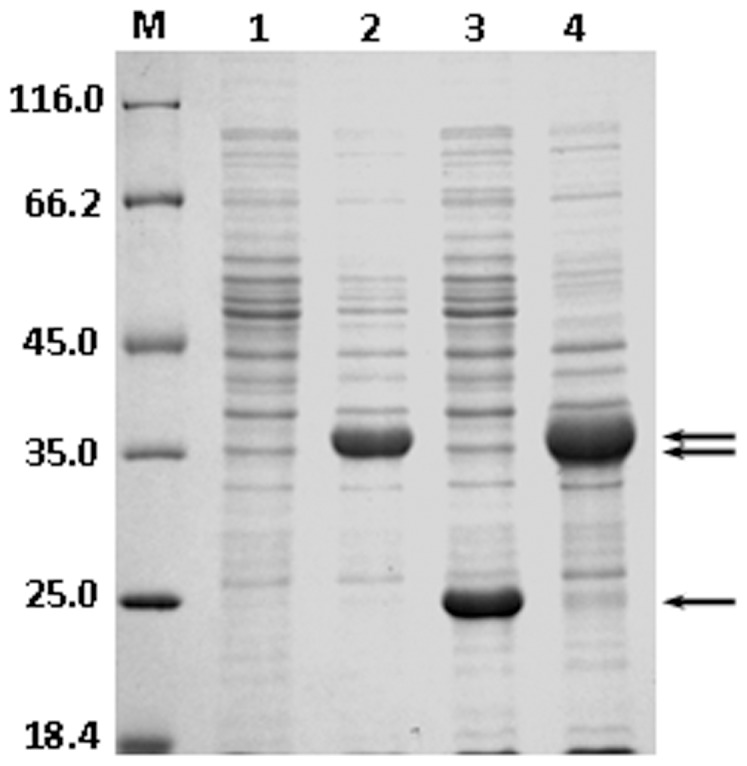
SDS-PAGE analysis of expression of *mmsB*, *PSEEN0851*, and *tsf* in *E. coli* JM109. The transformants were grown in LB at 37°C and induced by 1 mM IPTG. Lanes: M, molecular mass marker; 1, pQE-80L (as control); 2, pQE-*mmsB*; 3, pQE-*PSEEN0851*; 4, pQE-*tsf*. Arrowheads indicate the locations of recombinant proteins.

**Figure 4:**
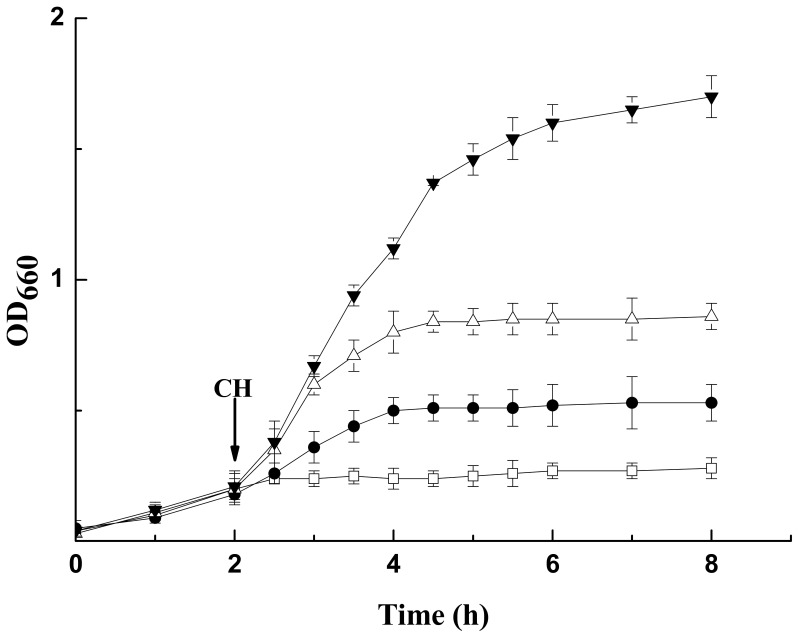
Cell growth of recombinant *E. coli* JM109 strains in the presence of 4% (v/v) cyclohexane. The recombinant strains were cultured at 37°C and cyclohexane (CH) was added when OD_660_ reached 0.2 (indicated by the arrow). □, pQE-80L (as the control); ▾, pQE-*mmsB*; •, pQE- *tsf*; △, pQE-*PSEEN0851*.

In order to validate the OST-related function of above three genes, recombinant plasmids harboring *mmsB, tsf*, and *PSEEN0851* were constructed and over-expressed in *E. coli* JM109 individually (Fig. 3). The recombinant strains were cultured in LBGMg medium till 0.2 OD_660_ when cyclohexane was added. As shown in Fig. 4, all 3 transformants exhibited higher cyclohexane tolerance than the control (*E. coli* JM109 carrying empty pQE-80L) when grown in the presence of 4% (v/v) cyclohexane. Especially for recombinant strain over-expressing *mmsB* gene, a stunning cell density of 1.70 OD_660_ was reached after 8 h. The expression of *PSEEN0851* and *tsf* also contributed to the higher OST of recombinant *E. coli*, rendering 0.58 and 0.25 increase in OD_660_ respectively. For the control strain, however, no appreciable growth was observed after the addition of cyclohexane.

**Figure 5:**
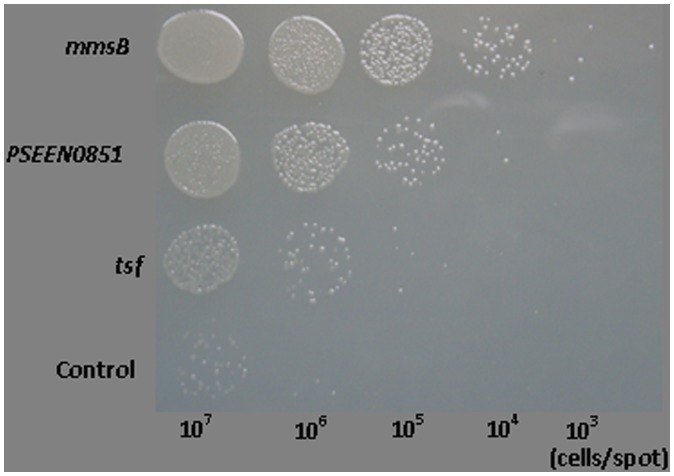
Colony formation of *E. coli* JM109 strains over-expressing *mmsB*, *tsf*, and *PSEEN0851* on LBGMg agar overlaid with decalin after 24 h incubation at 37°C. JM109 carrying empty pQE-80L was used as the control.

Colony-formation efficiency, a commonly used experiment to evaluate the OST of microbial cells [25], was also adopted in this study to examine the effect of three genes. Approximately 10^7^, 10^6^, 10^5^, 10^4^, and 10^3^ cells were dropped in the spots on agar plate which was then overlaid with decalin (Fig. 5). Similar as liquid cultivation in 4% cyclohexane, *mmsB* transformants exhibited the highest OST among three genes. The colony-formation efficiency of *E. coli* JM109 was markedly increased by over-expression of *PSEEN0851*. The expression of *tsf* also slightly increased the solvent tolerance of JM109. Therefore, three genes (*mmsB, tsf* and *PSEEN0851*) identified in 2-DE analysis of *P. putida* JUCT1 could be associate with the OST performance of other Gram-negative strains such as *E. coli*.

Furthermore, the potential oxidation or ring cleavage of cyclohexane might also contribute to the observed OST performance. In this study, the cyclohexane oxidation by *mmsB* transformants and *E. coli* JM109 (the control) was tested. Based on GC-MS analysis, cyclohexane was oxidized to cyclohexanol by both strains (Table 3). Compared with the control, lower cyclohexanol (0.206 g/L vs 0.431 g/L) and higher cyclohexane (0.695 g/L vs 0.452 g/L) levels were detected in the supernatant of *mmsB* transformants cell suspension. Apparently, 3-hydroxyisobutyrate dehydrogenase (encoded by *mmsB*) does not catalyze the oxidation or ring cleavage of cyclohexane. It is presumed that 3-hydroxyisobutyrate dehydrogenase, which exhibited prominent effect on enhancing OST of *E. coli*, could help keep a relative lower intracellular cyclohexane concentration by affecting cell membrane composition and accelerating organic solvent extrusion, and as a result, less cyclohexane was oxidized into cyclohexanol by cytoplasmic enzyme system of *mmsB* transformants.

**Table 3:** The oxidation of cyclohexane by *E. coli* strains.

	*E. coli* JM109 (pQE80L-*mmsB*)	*E. coli* JM109
Cyclohexane (g/L)	0.695±0.010	0.452±0.012
Cyclohexanol (g/L)	0.206±0.013	0.431±0.006

Incubation condition: 37°C for 8 h with cyclohexane (1%, w/v).

## Discussion

Adaptation of microorganisms to certain environment is a favorable method to obtain strain with improved phenotype. In this study, an OST strain *P. putida* JUCT1 capable of growing in the presence of 60% (v/v) cyclohexane as well as high concentration of other organic solvents was obtained after adapatation in cyclohexane. Adaptation of *E. coli* strains to organic solvents was also conducted, but all of them failed to grow in the presence of 2% (v/v) cyclohexane (data not shown).

As a powerful protein separation technique, 2-DE is widely used in the characterization of complex protein samples associated with certain phenotype. Matching protein spots between gels of similar samples could be quantified using software package such as PDQuest. In order to identify proteins associated with OST of *P. putida* JUCT1 and further understand its mechanisms, 2-DE was applied in this study to analyze its total cellular protein samples under different solvent conditions. Three proteins were identified to be important for solvent tolerance, specifically 3-hydroxyisobutyrate dehydrogenase, protein chain elongation factor EF-Ts, and isochorismatase superfamily hydrolase.

Both liquid cultivation and colony-formation experiments indicate that the expression of 3-hydroxyisobutyrate dehydrogenase (encoded by *mmsB*) conduced to the highest OST among three proteins identified. Enhanced expression of 3-hydroxyisobutyrate dehydrogenase under solvent stress could be an important way to produce more energy for extruding toxic organic solvents, because this enzyme plays an essential role in the catabolism of amino acids including valine, leucine and isoleucine. Additionally, the enzymatic mechanism and evolutionary origin of 3-hydroxyisobutyrate dehydrogenase is similar to that of 6-phosphogluconate dehydrogenase, and both of them belong to the 3-hydroxyacid dehydrogenase family [29], [31], [32]. In *E. coli*, the 6-phosphogluconate dehydrogenase encoding gene *zwf* belongs to the *mar-sox* regulon genes, which are important for the regulation of a number of stress response genes (several regulator genes such as *robA*, *soxS*, *marA* have similar functions), and the efflux pump such as AcrAB-TolC is under the control of stress response genes [20], [22]. In *P. putida*, similar mechanism (such as Mex efflux systems) also exists [14], [33]. It is therefore speculated that 3-hydroxyisobutyrate dehydrogenase is involved in the OST regulation in *P. putida* and dedicates to the enhanced solvent tolerance.

In *E. coli*, isochorismatase catalyzes the hydrolysis of isochorismate to 2,3-dihydroxy-2,3-dihydrobenzoate and pyruvate, although the specific function of isochorismatase superfamily hydrolase in *Pseudomonas* is unknown [30]. Amino acid sequence alignment shows that the similarity between isochorismatase superfamily hydrolase in *P. putida* JUCT1 and *E. coli* is more than 94%. The two enzymes may share similar activity as well as OST-related functions by unknown mechanisms.

Protein chain elongation factor EF-Ts (encoded by *tsf*) is another protein identified from 2-DE images based on its intensity discrepancy. Our result shows that the OST of *E. coli* was slightly enhanced by the over-expression of protein chain elongation factor EF-Ts (encoded by *tsf*), suggesting that EF-Ts might assist in the expression of certain stress-response proteins to improve the solvent tolerance. Similar result has been reported in toluene tolerance of other *P. putida* strains [26], [27].

The OST mechanisms in Gram-negative bacteria are not completely understood so far. Despite their different taxonomy, several similar mechanisms in overcoming the destructive effects of organic solvents have been reported in *Pseudomonas* and *E. coli* strains, such as efflux pump and changing of membrane structure [11], [14], [19], [21]. In this study, proteomic analysis was proven to be an effective method in identifying OST-related proteins. The OST-related functions of three genes from *P. putida* were confirmed by their recombinant expression in *E. coli*. Homologs of these genes in other bacteria might also involve solvent-tolerance mechanism of their own. This study demonstrates a feasible approach to explain microbial OST mechanisms, and provides molecular basis to construct OST microorganisms in industrial applications such as whole-cell biocatalysis, alcohol production.

## Materials and Methods

### Bacterial Strains and Plasmids

*Pseudomonas putida* JUCS capable of growing in the presence of 1% (v/v) toluene was isolated from wastewater, and was identified as *Pseudomonas putida* based on morphological and biochemical characterization, as well as 16S rRNA sequence analysis. *P. putida* JUCS is deposited at China General Microbiological Culture Collection Center (CGMCC) under the accession number CCTCC M 2011442. *P. putida* JUCT1 was obtained by gradient adaptation in cyclohexane from *P. putida* JUCS. *E. coli* JM109 (*traD*36, *proAB*+, *lacIq*, *lacZ ΔM15*) is an organic-solvent sensitive strain and was used as the host strain. The *cis*-repressed pQE-80L *kan* vector was purchased from QIAGEN Co. (Germany).

### Growth and Adaptation Conditions

*Pseudomonas putida* JUCS cells was grown in nutrient broth medium (consisting of 1% (w/v) peptone, 0.3% beef extract, 0.5% NaCl, pH 7.0) under 30°C in a shaker. To maintain the solvent tolerance, 1% (v/v) toluene was added to the nutrient broth medium. *E. coli* JM109 cells was grown in LB medium at 37°C. Cell growth was monitored by measuring optical density at 660 nm.

*P. putida* JUCS was initially cultured under 1% (v/v) toluene, and transferred to nutrient medium containing 5% (v/v) cyclohexane. Then the culture was transferred to nutrient medium agar plate (11 cm) overlaid with 500 µL cyclohexane. The colonies obtained from agar plate were further cultured under higher cyclohexane concentration of 10% (v/v), followed by isolating from agar plate overlaid with 1 ml of cyclohexane. The cultivation for each transfer step was at 30°C for 12 h. As a result of adaptation in medium supplemented with escalating cyclohexane in each transfer; a cyclohexane-tolerant *P. putida* strain, referred as *P. putida* JUCT1, capable of growing in the presence of 60% (v/v) cyclohexane was finally obtained.

### Extraction of Total Cellular Protein

Microorganism protein sample was prepared as follows. *P. putida* JUCT1 was cultivated aerobically to mid-exponential phase (0.8 A_660_) at 30°C in the presence of 60% (v/v) cyclohexane, and the strain grown in nutrient medium was used as control.

Cells were harvested by centrifugation at 6,000 × g and 4°C for 5 min, and the cell pellet was washed three times with cold water to reduce the ion concentration. Then the cell pellet was resuspended in ice buffer (0.167 g wet cells/ml) containing 8 M urea, 2 M thiourea, 65 mM DTT, 4% (w/v) CHAPS, 40 mM Tris-base and 0.001% (w/v) bromophenol blue. The cells were disrupted by ultrasonication (300 w, pulse 1 s, pause 3 s for 30 min) in ice bath. The cell-free extract was obtained by centrifugation at 20,000 × g and 4°C for 15 min, and was used as total cellular protein for further 2-DE analysis. The protein concentration was determined by RC-DC Protein Assay Kit (Bio-Rad).

### 2-D Electrophoresis

In our preliminary experiment, IPG 3-10 strips (7 cm, GE Healthcare, Pittsburgh, PA) were used to determine zone allocation of protein samples. The result indicates that most proteins locate on the pH 4 to 7 region of the IPG strips (image not shown). The total cellular protein samples were then separated by IPG 4−7 strips (13 cm, GE Healthcare). Briefly, the Immobiline IPG Drystrip was hydrated with 400 µg of total cellular protein sample in 250 µl of rehydration buffer which contains 8 M urea, 2 M thiourea, 65 mM DTT, 4% (w/v) CHAPS, 0.2% (v/v) IPG buffer (pH 4–7), 40 mM Tris-base and 0.001% (w/v) bromophenol blue for 16 h at 20°C. The isoelectric focusing (IEF) was carried out using Ettan IPGphor 3 system (GE Healthcare) at 20°C as follows: 50 V for 30 min, gradient to 150 V for 30 min, gradient to 500 V for 1 h, gradient to 1,000 V for 2 h, gradient to 4,000 V for 3 h, gradient to 8,000 V for 3 h, holding at 8,000 V, 40,000 V/h, and for the total of 67,400 Vh. After IEF, the IPG strips was incubated in equilibration buffer I for 15 min, then with the equilibration buffer II for 15 min. Equilibration buffer I contains 8 M urea, 2% (w/v) SDS, 0.375 M Tris-HCl (pH 8.8), 20% (v/v) glycerin and DTT (20 mg ml^−1^); and equilibration buffer II is the same as I except iodoacetamide (25 mg ml^−1^) is used instead of Dithiothreitol (DTT). The IPG strips were then washed twice with ultrapure water and transferred onto 12% SDS-polyacrylamide gel. The second dimension electrophoresis was conducted at 10°C with two steps: step 1, 2 w gel^−1^ for 1 h; step 2, 8 w gel^−1^ for 4 h.

After 2-DE was completed, the 2-D gel was stained for 2 h with coomassie brilliant blue dye and de-stained in solution containing 10% (v/v) methanol and 10% (v/v) acetic acid. The de-stained gel images were obtained by ImageScanner III (GE Healthcare, Pittsburgh, PA) and analyzed by PDQuest™ 2-D Analysis Software (Bio-Rad). The 2-DE experiment was conducted with three biological replicas.

### Protein Identification by MALDI-TOF/TOF

Protein spots were excised from 2-D gel, and subjected to in-gel digestion. Peptides from trypsin digestion were analyzed using 4800 Plus MALDI TOF/TOF™ Analyzer (Applied Biosystems, USA). Combined peptide mass fingerprinting PMF and MS/MS queries were performed by using the MASCOT search engine (Matrix Science, Ltd.) embedded into GPS-Explorer Software (Applied Biosystems) against database NCBI Bacteria (320879). The following settings were used: mass accuracy was ±100 ppm, MS/MS fragment tolerance was 0.8 Da, carbamidomethyl and oxidized methionine were set as fixed and variable modifications respectively, one missed cleavage was allowed in trypsin cleavage. A GPS-Explorer protein confidence index of ≥95% was used for further manual validation.

### Cloning and Expression of *mmsB*, *tsf*, *and PSEEN0851*

Chromosomal DNA of *P. putida* JUCS was prepared and used as the template to amplified the genes of *mmsB* (GenBank accession number: JC7926), *tsf* (GenBank accession number: CAK16908), and *PSEEN0851* (GenBank accession number: CAK13762). A set of primers were used as summarized in Table 4. The *cis*-repressed pQE-80L vector and PCR product were digested with restriction enzyme suitably. After ligation, the recombinant plasmid was transformed into *E. coli* JM109 by heat shock method. The recombinant plasmids were verified by double-enzyme cleavage, and the expression of recombinant protein was analyzed by SDS-PAGE after 1 mM IPTG induction.

**Table 4:** Primers for PCR amplification of genes *mmsB*, *tsf, and PSEEN0851*.

Primer	Sequence (5′→3′)^a^	Length (nt)
*mmsB*-F	ATCGGGATCCATGCGTATTGCATTCATTGG	30
*mmsB*-R	CCCCAAGCTTTCAATCCTTCTTGCGATACC	30
*tsf*-F	CGCGGATCCATGGCAGCAATTACTGC	26
*tsf*-R	CGCAAGCTTTTACTGCTTGGCGGCAG	26
*PSEEN0851*-F	CGCGGATCCATGGCTTTCCACTACAA	26
*PSEEN0851*-R	AACTGCAGCTACTTGGCCAGCTTGCTG	26

a The underlined sequences are the restriction enzymes sites.

### OST Assay

In our preliminary tests, *E. coli* JM109 is an organic-solvent-sensitive strain, and the cell growth was completely inhibited by as low as 2% (v/v) of cyclohexane (Log *P_ow_* = 3.2). To examine the effect of the above three genes on solvent tolerance, the transformants were grown in LBGMg medium (consisting of LB medium, 0.1% glucose (w/v), 10 mM MgSO_4_) at 37°C, and cyclohexane was added to the medium in a final concentration of 4% (v/v) when OD_660_ reached 0.2. The mixture was further cultured at 37°C and monitored by cell density (OD_660_).

The agar medium analysis was used to measure the colony-forming efficiency. The cells were grown in LBGMg medium until the cell density (OD_660_) reached 1.0. A series of 10-fold dilutions of cultures were prepared and 4 µl of the each cell suspension was spotted on the LBGMg agar medium. The LBGMg medium was then overlaid with organic solvent and incubated at 37°C for 24 h.

Furthermore, the potential oxidation (or ring cleavage) of cyclohexane by *E. coli* strains was determined in potassium phosphate buffer (0.2 mol/L, pH 7.0) containing cyclohexane (1%, w/v), 1 g of wet cells, glucose (5%, w/v) with a total volume of 10 ml. After incubation at 37°C for 8 h, the cell suspension was centrifuged, and the supernatant was extracted with ethyl acetate, then the organic phase was subjected to GC and GC-MS analysis. The products were analyzed using a Varian 3900 gas chromatography (Palo Alto, CA) equipped with a PEG-20,000 column (30 m×0.32 mm×0.4 µm) using an flame ionization detector. The oxidation products of cyclohexane were identified by an Agilent 6890-5973N GC/MS equipped with a same column (Santa Clara, CA).
